# Conceptual Spaces for Conceptual Engineering? Feminism as a Case Study

**DOI:** 10.1007/s13164-023-00708-7

**Published:** 2023-12-19

**Authors:** Lina Bendifallah, Julie Abbou, Igor Douven, Heather Burnett

**Affiliations:** 1 Institute of Philosophy, University of Leuven, Leuven, Belgium; 2 Dipartimento du Culture, Politica e Società, University of Turin, Turin, Italy; 3 IHPST, CNRS, Panthéon-Sorbonne University, Paris, France; 4 Laboratoire de Linguistique Formelle, Paris Cité University, Paris, France

## Abstract

Recently, there has been much research into conceptual engineering in connection with feminist inquiry and activism, most notably involving gender issues, but also sexism and misogyny. Our paper contributes to this research by explicating, in a principled manner, a series of other concepts important for feminist research and activism, to wit, feminist political identity terms. More specifically, we show how the popular Conceptual Spaces Framework (CSF) can be used to identify and regiment concepts that are central to feminist research, focusing especially on feminism in France. According to the CSF, concepts can be represented geometrically, as regions in similarity spaces. A particular strength of the CSF framework is its empirically-focused methodology, which allows researchers to infer the boundaries of concepts from empirical data, thus eliminating the need to strongly rely on intuitions about meanings. This is shown to be especially valuable for the explication of concepts relating to feminist political identity, given that the intuitions of feminist scholars and activists about what would appear to be core concepts in the area tend to be poorly aligned or even conflicting. We report the results from an empirical categorization study conducted among French feminists and show how they support the view that the CSF can contribute to both the conceptual engineering project and our understanding of the structure of social reality.

## Introduction

This paper argues that the *Conceptual Spaces Framework* (CSF; e.g., Shepard 1964; Nosofsky 1986, 1987, 2011; Gärdenfors 2000, 2014) can be helpful to philosophers engaged in the project of conceptual engineering. While noting that the meaning of *conceptual engineering* is not completely fixed, Cappelen and Plunkett (2020, p. 3) define it as “the assessment and improvement of concepts,” or, more broadly, as involving “(i) The assessment of representational devices, (ii) reflections on and proposal for how to improve representational devices, and (iii) efforts to implement the proposed improvements.” While this project has attracted a fair amount of attention in recent years, it is closely connected to Carnap’s work on *explication* (Cappelen 2018; Cappelen and Plunkett 2020; Dutilh Novaes 2020). In a frequently cited passage, Carnap describes the process of explication as follows:

The task of making more exact a vague or not quite exact concept used in everyday life or in an earlier stage of scientific or logical development, or rather of replacing it by a newly constructed, more exact concept, belongs among the most important tasks of logical analysis and logical construction. We call this the task of explicating, or of giving an *explication* for, the earlier concept; this earlier concept, or sometimes the term used for it, is called the *explicandum*; and the new concept, or its term, is called an *explicatum* of the old one. (Carnap, 1947, p. 7f)

Explication and, by extension, conceptual engineering have been viewed as fundamental to much work in philosophy of science, mathematics, and logic (Kuipers 2007; Justus 2012; Brun 2016, 2020; Dutilh Novaes and Reck 2017; De Benedetto 2020). More recently, many philosophers have also attempted to apply these processes to the social domain with the hope that, in addition to arriving at a better understanding of various social concepts, the results of explication/conceptual engineering could have a part to play in social movements like feminism and anti-racism. For example, in her now classic article “Gender and race: (What) are they? (What) do we want them to be?,” Haslanger (2000, p. 33) lays out a research program for (what she calls) ameliorating the concepts of gender and race with the hope that “a theory offering an improved understanding of our (legitimate) purposes and/or improved conceptual resources for the tasks at hand might reasonably represent itself as providing a (possibly revisionary) account of the everyday concepts”. The ideas in Haslanger’s paper have inspired much research into the explication/engineering/amelioration of concepts central to feminist inquiry and activism, most notably *gender* (following Haslanger; see Witt 2011; Jenkins 2016; Díaz León 2020, among many others), but also *sexism* and *misogyny* (Manne , 2017).

We aim to contribute to this growing body of work in analytic feminism by helping to explicate a series of other concepts which are important for feminist research and activism, viz., feminist political identity terms. More specifically, we show how the CSF can be applied to the domain of feminism.^1^ The CSF is currently one of the main frameworks in cognitive science for studying concepts and their role in reasoning. The core idea of this framework is that concepts can be represented geometrically, as regions in so-called conceptual spaces. A particular strength of the CSF framework is its empirically-focused methodology, which, at least ideally, allows researchers to infer the boundaries of concepts from empirical data, thus eliminating the need to *strongly* rely on philosophers’ or other people’s intuitions about meanings.^2^ This is shown to be especially valuable for the explication of concepts relating to feminist political identity, given that, as will be seen, feminist scholars and activists tend to assign a variety of meanings to the terms central to their discourse, sometimes in ways that create conflict. The primary aim of this paper is therefore to delineate concepts associated with feminist political identity terms by using CSF’s empirically-based methodology.

A secondary aim concerns the CSF. While this framework has been shown to be very successful when applied to perceptual concepts, giving rise to well-validated color spaces, taste and olfactory spaces, auditory spaces and shape spaces, and more (see Kaipainen et al., 2019), its empirical scope (i.e., the kinds of conceptual domains that it has been used to analyze) has so far remained fairly limited.^3^ This has not been due to researchers’ lack of imagination, but rather to a concern that, once we step away from basic perceptual domains, an approach based on conceptual spaces may no longer be enlightening or even appropriate. This worry is succinctly summed up by Douven et al. (2013, p. 138), who say, concerning the CSF,

And it must be admitted that, while this approach [i.e., reliance on the CSF] works very well for perceptual concepts, it does not obviously apply to more complex and abstract concepts. It may be a matter of time before the theory can satisfactorily deal with the latter kinds of concepts as well. Or, given that it is not a priori that all conceptualization must eventually be accounted for in the same terms, non-perceptual concepts may require some altogether different approach.

Also in connection with the idea of explicating the concept of *explication* in terms of conceptual spaces, De Benedetto (2020, p. 855), makes basically the same point when he says that “The adequacy of conceptual spaces representation of concept formation and manipulation has been empirically tested for many types of concepts $\ldots$, but the exact scope of applicability of the theory is still unclear.” The secondary aim of our paper is therefore to help answer the following open question: *Once we leave behind the cognitively basic domains and move to more complicated social ones, can the framework, and its experimental methodology, still be fruitfully applied?* Our answer to this question will be generally positive.

The paper is organized as follows: In Section 2, we present our main empirical focus, to wit, feminist political identity terms, in particular those current in debates among French feminists. We argue that the concepts related to feminist identity are instances where explication and engineering will be useful. As we will show, although they are frequently used and structure both scholarly activities and activism in France, many feminists express reservations about the ways in which they are employed. We therefore propose that conceptual engineering has a role to play here, and turn to conceptual spaces to help this process. In Section 3, we start by observing that the use of spatial representations in sociology has a long history, and we suggest that insights from social spaces provide a way to apply the CSF to a complex social domain like feminism. Then, in Section 4, we combine social and conceptual space methodologies to construct conceptual spaces for French feminist political identity terms, based on an empirical categorization study with data from 81 French feminist participants. We argue that the representations obtained through the CSF can be taken to provide explications of the feminist identity terms commonly used by scholars and activists. Section 5 concludes with an evaluation of the engineered concepts and, more generally, the application of CSF outside the perceptual domain.

## Feminist political identity terms

Given the diverse range of approaches and actions encompassed by feminism, multiple perspectives and representations of women and their interests have flourished within feminist scholarship, activism, and various social domains. As Bereni (2012) points out, feminist struggles have emerged across a wide spectrum of social and political spheres. These include political parties, social and political movements, elite professions, intellectual realms, and public institutions.

In France in the 1960s, Antoinette Fouque and Monique Wittig played a pivotal role in the emergence of the *Women’s Liberation Movement* (MLF). However, as the movement progressed, theoretical divergences began to surface, including disagreement regarding the term *feminism* itself. This led to a significant fracture within the MLF, resulting in the formation of two distinct currents: differentialist feminism, spearheaded by Fouque (e.g., Fouque, 1995) and materialist feminism, represented by Wittig (e.g., Wittig, 1980). Differentialist feminism posits that inherent differences exist between men and women and argued for a reassessment of so-called women’s values.

On the other hand, **materialist feminism** focuses on the material conditions and economic factors that contribute to gender inequality and oppression. It emphasizes the ways in which capitalism and patriarchy intersect to shape *women* as a class constituted by social relations of oppression and exploitation (Delphy, 2008). They argue that women’s oppression is rooted in the structures of capitalism, which commodifies labor and creates economic disparities. Materialist feminists advocate for economic justice, equal access to resources and opportunities, and the transformation of societal structures to address the underlying economic factors that perpetuate gender inequality. They argue that true gender equality can only be achieved by challenging and transforming the capitalist systems that perpetuate women’s oppression.

Very early, activists and scholars challenged the unity and homogeneity of women as a group by showing that it is crossed by inequalities related to class, race, and sexuality. One notable contribution in this regard was made by Crenshaw (1989) who introduced the concept of **intersectionality** which refers to a tool for analysis that aims to theorize the convergence, co-constitution, imbrication or interwovenness of systems of oppression and the unique configuration of multi-oppressed subjects.^4^ Crenshaw’s work focuses on domestic violence and shed light on the often invisible isolation experienced by African American women due to the impact of racism and sexism. She notes that agencies serving women who have experienced intimate partner violence fail to consider how factors like poverty and immigration status fundamentally shape specific needs of certain women. Immigrant women, for instance, may face language barriers, fear of deportation, and other forms of discrimination that are not taken into account. Intersectionality, as a framework, acknowledges that individuals and groups can simultaneously experience multiple forms of disadvantage and highlights the need to consider the interconnected nature of these intersections of social features as well as the complex power dynamics that arise from their combination. It has been influential in various fields and has helped to shed light on the limitations of an approach that only focuses on one aspect of social identity, allowing a more nuanced understanding of how different systems of oppression intertwine and impact people’s lives.

The term **queer** has multiple meanings. In contemporary usage, “queer” is employed as an umbrella term that encompasses diverse sexual orientations and gender identities. As an academic field, “queer theory” refers to a framework that has emerged in the late 20th century. It critically examines the relationship between sexuality and gender, challenges normative understandings of sexual and gender identities while questioning heteronormative structures that underpin the gender binary and sexual norms. At its core, queer theory rejects that sexuality and gender are determined by biology and emphasizes the fluidity of these identities. Queer theorists explore how norms and discourses around sexuality and gender are used to marginalize and exclude non-conforming individuals and communities (e.g., De Lauretis, 1991).

The terms described above (*materialist*, *intersectional*, *queer*, etc.) were introduced (in their relevant meaning) in feminist scholarship, originally applied to analyses, ideas, or approaches. However, it has now become common to apply such terms to people, either when self-identifying or when categorizing someone else. It is this “identity term” use that is the empirical focus of our paper, and we will first illustrate it through examples from the *Cartographies linguistiques du féminisme* (CaFé) corpus (Abbou and Burnett , 2022a), which is a corpus of one hundred interviews with feminist activists and scholars in Paris, Montréal, and Marseille realized in 2021 and 2022. In this paper, we focus on the Parisian corpus (43 interviews). As shown in the examples in (1)–(3), terms like *queer* “queer,”^5^*matérialiste* “materialist,” and *intersectionnel(le)* “intersectional,” can all be used in identity statements (e.g., *je suis queer/matérialiste/intersectionnelle* “I am queer/materialist/intersectional”), which directly qualify people.

1. **Speaker 9:** alors moi pour le coup **je me sens queer, je me range dans les queer**. J’utilise le terme qui est un militantisme et qui pour moi recouvre en fait toutes les réalités, et qui est a une vision pour le grand public souvent paillettes je pense.^6^
2. **Speaker 36:** avec la fac où la plupart **des gens étaient matérialistes** et tout, j’ai plus un peu appris ça et ça m’intéresse de plus en plus, et beaucoup plus maintenant, donc je sais pas si je me dirais féministe matérialiste, ou je me dirais pas queer en tous cas ça c’est sur.^7^
3. **Speaker 18:** [describing her feminism] inspiré de la théorie queer mais des courants qui essaient de faire le lien entre le queer et le matérialisme quoi. Ce serait un peu dans ça et intersectionnel, enfin, qui se veut intersectionnel quoi, parce que c’est un peu facile de dire “**je suis intersectionnelle**” mais mon féminisme se voudrait queer et intersectionnel.^8^

The original transparent meanings of these adjectives applied to humans were undoubtedly related to the theoretical current, that is, “one who adopts a materialist/queer/intersectional analysis of phenomenon X,” but the meanings of these identity terms has since broadened to mean something more general (see, e.g., Bilge, 2015 or Rice et al. 2019).

Feminist concepts that were created by scholars are used and spread across political and social movements, as well as in the media and professional spheres. As discussed by Hardin (1992) and Puig de la Bellacasa (2013), however, the different social spheres are often not easy to distinguish from one another, often by design. As the CaFé corpus shows, many of the interviewees, who include academics and activists in areas related to feminism and gender in Paris, identify a lack of consensus about the new uses of terms like *matérialiste*, *queer*, or *intersectionnelle*, either because they feel they do not know what they mean well enough or because they consider that other people misuse them. This is despite actively applying these terms to themselves and others, and structuring important aspects of their activism (meetings, associations, parties, and so on) around them.

For example, in (4), speaker 36 describes how, despite participating in “queer” activism in Paris, she does not quite know what the word means (in this particular use):

(4) **Speaker 36:** quand j’ai rencontré à Lyon des gens qui s’intéressaient au rapport entre **matérialisme** et **queer**, et du coup on faisant souvent des débats $\ldots$ parce que je fréquente un milieu qui se définit comme **queer**, des gens qui se définissent de plus en plus comme **queer**. J’ai un peu du mal à voir ce que ça recouvre exactement.^9^ Likewise, speaker 21 confesses that she identified as “matérialiste” for a long time, while all the while feeling like she did not really know what the word meant:
(5) **Speaker 21:** Pendant longtemps je me suis définie comme **féministe matérialiste** sans trop savoir ce qu’il y avait derrière à part ce qu’on en disait entre camarades.^10^ Furthermore, many of the speakers in the CaFé corpus consider that *intersectionnel(le)*, when used as an identity term, is ambiguous at best, and, at worst, almost empty of all meaning. These sentiments are expressed by speakers 32 (6) and 22 (7), when asked about expressions like *Je suis intersectionnelle*.
(6) **Speaker 32:** Moi je pense que j’ai mis au coeur de mon féminisme les enjeux de de classes et de race, mais j’ai l’impression que tout le monde dit ça mais que personne met la même chose derrière $\ldots$ il y a quelques années j’aurais donné tous les termes, en fait, j’aurais dit “mon féminisme est **matérialiste**, **intersectionnel**, **décolonial** etcetera,” et en fait je me rends compte que, quand on utilise ces termes là, on c’est aussi une manière de masquer ce qu’on pense. **Interviewer:** d’entretenir le flou un peu ouais ouais ouais.^11^
(7) **Speaker 22:** J’ai l’impression que c’est un terme qui est revendiqué parce qu’il est évident que le féminisme doit être **intersectionnel** pour les féministes, mais en fait il est instrumentalisé pour éviter de penser les relations de domination qui sont pas euh nécessairement liées au genre. Donc son utilisation dans ce sens-là, m’embête un peu. C’est un peu de dire euh “bah moi je suis féministe du coup j’ai pas besoin de penser à aux relations que j’ai avec –” enfin tu vois $\ldots$ de dire “bah en fait **nous on est intersectionnelles**, donc euh voilà c’est pas un problème si t’es racisé et tout” et sans vraiment réfléchir aux relations de domination qui existent. Donc ouais c’est un terme dans son utilisation qui est un peu creux, mais bien-sûr pour moi quelque chose qui est absolument essentielle.^12^ Finally, different uses of feminist political identity terms can even give rise to conflict. This is illustrated by speaker 1 (8), who recounts how she and an afrofeminist activist were in disagreement over whether an association which had members who were not afro-descendants could identify as *intersectionnelle*.
(8) **Speaker 1:** On avait dit qu’on était une asso **intersectionelle** et qu’en fait les afroféministes, il y a une meuf militante qui nous a écrit pour nous dire “oui vous pouvez pas dire que c’est **intersectionnel** parce que c’est un mot réservé aux afro-descendants” mais bon apparemment si tu l’utilises comme un adjectif en fait ça sous-entend que t’es une asso justement afroféministe quoi.^13^

As it appears from these quotations, meanings related to French feminist political identity terms can be subject to multiple interpretations and meanings, which can lead to conflicting perspectives among feminist activists. At the same time, they appear ambiguous to members of this community. This suggests that these terms may be good candidates for further clarification using tools from the CSF. To this end, we propose a method that aims to identify a kind of best compromise (in a sense that will become clear further on). Our approach is meant to be *conservative* by deviating as little as possible, on average, from the concepts currently in use. At the same time, it is guided by certain other virtues, such as optimal design principles, which will be discussed below.

## Social spaces as conceptual spaces

Before we present the results of our engineering process, we describe the main properties of the CSF and explain why we think it may be an important tool for conceptual engineers.

### Conceptual spaces for conceptual engineering?

Philosophers have long held the view that concepts, broadly conceived as the mental correlates of predicates, are individuated by the criteria an item has to satisfy for it to fall under a given concept. For instance, to fall under the concept of human being is, on this view, to be a mammal, to be rational, to have four limbs, and to satisfy some further criteria. However, psychological research has found little support for this view of concepts. To determine whether an item falls under a given concept, people appear to judge how similar the item is to one or more other items that are already known to fall under the same concept—a most typical item, or prototype, according to some researchers, or a number of salient items, according to exemplar theorists—instead of going through a checklist of criteria. This observation led to the development of a mathematical framework in which concepts are modeled geometrically, as regions in *similarity spaces*.

Similarity spaces are one- or multi-dimensional metric spaces whose dimensions represent fundamental attributes that items can have to a greater or lesser degree. We can thus map items onto points in a given space, according to the degrees to which the items have the attributes represented by the space’s dimensions, and distances between items in the space are to be interpreted as representing dissimilarities: the greater the distance, the more dissimilar the items are in the respect modeled by the space; conversely, the closer the items are to each other, the more similar they are.

There are a number of ways of arriving at a similarity space. The most common one starts by collecting judgments concerning the similarity of particular items, where the similarity is again in the respect one aims to model (so, for instance, how similar in shape items are, if one wants to construct a shape space); sometimes researchers use instead confusion probabilities, indicating the likelihood that two distinct stimuli are mistakenly judged to be identical when consecutively flashed to participants, or correlation coefficients among items, indicating how strongly items co-vary in certain designated respects. These data are then often transformed into a matrix representing the similarities among stimuli as distances, which in turn serve as input for some statistical dimension-reduction technique, most commonly multidimensional scaling (MDS; other techniques sometimes used include principal component analysis and non-negative matrix factorization), which outputs a one- or multi-dimensional similarity space in which the items under consideration (i.e., the stimuli that were used to elicit the similarity judgments or the confusion probabilities) are represented as points, and where the distance between any given pair of points is meant to reflect how similar the corresponding items are to each other in the respect the space is supposed to represent (Borg and Groenen , 2005).

In this procedure, the aim is not just to arrive at *some* spatial representation of the input data, but to arrive at one that (i) is *low-dimensional*, ideally (for the purposes of visualization) with no more than three dimensions; (ii) fits the data *well*, usually measured in terms of “stress,” with lower stress values indicating more adequate representations of the input data (i.e., distances in the space more accurately reflect similarities); and (iii) has dimensions we can *interpret*, in that with each dimension we can plausibly associate a fundamental attribute the items under consideration can be said to have to some degree (Borg and Groenen , 2005).

There is no guarantee that the result of the procedure will be satisfactory; for instance, there may simply be no low-dimensional space whose associated stress value is acceptable. But if the result *is* satisfactory, then that still only gives us a similarity space, and not a conceptual space. There is again more than one way to obtain a conceptual space from a similarity space. A prominent one relies on *prototype theory* in combination with the mathematical technique of *Voronoi tessellations*. According to the former, concepts have a “most representative instance,” whether real or idealized, which is its *prototype* (Rosch , 1973). And a Voronoi tessellation of a given space is a partitioning of that space into disjoint cells such that each cell is associated with exactly one so-called generator point and contains all and only those points in the space that lie at least as close to that cell’s generator point as they lie to any other cell’s generator point (Okabe et al. , 2000). Formally, where *S* is a similarity space, $\delta$ a distance metric defined on *S*, and $\langle p_1,$...$,p_n\rangle$ a sequence of pairwise distinct points in *S*, the region

$$\begin{aligned} v(p_i) , :=\,\Big \{p \, | \, \delta (p,p_i) \leqslant \delta (p,p_j),\text {for all}\ j\in \{1,\ldots ,n\}\ \text {with}\ j\ne i\Big \} \end{aligned}$$

is the *Voronoi polygon/polyhedron associated with* $p_i$, and collectively the members of $\left\{ v(p_i)\right\} _{1\leqslant i\leqslant n}$ constitute the *Voronoi diagram generated by*$\langle p_1,$...$,p_n\rangle$ (for details, see Okabe et al. (2000, ch. 2). Given a similarity space, then, we first locate in it the prototypes of the various concepts we are aiming to represent and then use these as the generator points for a Voronoi tessellation of the space, which gives us a conceptual space.

There are other ways to build a conceptual space on top of a similarity space. One that we will use in this paper recruits a suitable clustering algorithm known from the machine learning literature. There are many such algorithms; however, all have the purpose of partitioning a collection of items into different clusters such that within-cluster similarity (how similar to each other items within a cluster are) and across-cluster *dis*similarity (how dissimilar items in different clusters are) are jointly maximized. Different algorithms formalize these notions in somewhat different ways. For instance, where *x* and *y* range over a set of items, *P* is a partitioning of *S*, and sim is a similarity relation defined on that set, with $0\leqslant \text {sim}(x, y)\leqslant 1$, a standard definition of the within-cluster similarity of *P* is this:

$$\begin{aligned} S(P):= {\sum }_{x,y:P(x)=P(y)}\text {sim}(x,y), \end{aligned}$$

while an equally standard definition of the across-cluster dissimilarity of *P* is this:

$$\begin{aligned} D(P):= {\sum }_{x,y:P(x)\ne P(y)}\bigl (1 - \text {sim}(x,y)\bigr ). \end{aligned}$$

Given these or similar definitions, clustering algorithms try to find some partitioning *P* which maximizes the sum of within-cluster similarity and across-cluster dissimilarity.

Among the best known algorithms of this kind are *k*-means clustering (MacQueen , 1967) and Partitioning Around Medoids (PAM; Kaufman and Rousseeuw, 2009), the latter being a variant of the former that is known to be more robust in that it is less sensitive to outliers in the data. This is why, in this paper, we use PAM. An important note on clustering algorithms is that they do not require any information about prototypes as input, which makes them preferable when no such information is available or when we are dealing with concepts that do not possess prototypes.^14^ It is further to be noted that, nonetheless, the algorithm produces a Voronoi tessellation of the given similarity space, not on the basis of a set of prototypes, but by starting to place more or less randomly a pre-specified number of *k* so-called centroids (in the case of *k*-means clustering) or medoids (in the case of PAM) in the space, let those generate a Voronoi tessellation, calculate the within-cluster similarity and across-cluster dissimilarity corresponding to that tessellation, and then use an optimization algorithm to iteratively find better and better placements of the centroids/medoids, until the process converges (that is, further iterations do not lead to an increase in the joint within-cluster similarity and across-cluster dissimilarity; see Kaufman and Rousseeuw, 2009).

Whichever of the aforementioned methods is applied, concepts come out as *regions* in similarity spaces.^15^ For instance, the concept of blueness comes out as a region in color similarity space (CIELAB or CIELUV space; see Fairchild, 2013); smells are regions in olfactory space (Castro et al. , 2013); the concept of being a vase is a region in some shape space (e.g., Douven, 2016); and so on.

If concepts are represented by regions in similarity spaces, the question arises whether any region in a similarity space does, or at least could, represent a concept. Gärdenfors (2000) has answered this question in the negative, at least insofar as we are interested in *natural* concepts, that is, concepts that do or could occur in our thinking and communication. His proposal was that only *convex* regions are suited to represent such concepts,^16^ where a region is convex if, and only if, for any two points in the region, the line segment connecting those points lies in its entirety in the region as well. Gärdenfors (2000, p. 70), conceives of this convexity requirement as “a principle of cognitive economy; handling convex sets puts less strain on learning, on your memory, and on your processing capacities than working with arbitrarily shaped regions.” As Douven and Gärdenfors (2020) observe, however, convexity alone is not enough to single out natural concepts; for instance, there are infinitely many convex regions of color space that clearly do not correspond to any natural color concepts.^17^ These authors therefore propose a number of further constraints on naturalness, which—they argue—are also supported by considerations having to do with cognitive economy. For instance, they propose that a conceptual space should be *informative* in that it should allow users to make fine-grained distinctions in all parts of the similarity space on which it is built. As they also note, however, given that humans have limited memory capacity, informativeness should be weighed against the system being *parsimonious*, so as to tax our memory no more than is required for users of the space to function properly. In short, Douven and Gärdenfors propose that natural concepts are represented by the cells of an *optimally partitioned* similarity space, where an optimal partition is one that does best, on balance, in satisfying their various constraints.

Under the assumption that the aims of conceptual engineering are broadly in line with those of Carnapian explication, we can consider Carnap’s (1962a) criteria for successful explication and ask whether the CSF could be a useful tool for conceptual engineers. These criteria have been analyzed at length in the philosophical literature, but here we present Cappelen’s (2018) summary, which he gives in a discussion of the relevance of those criteria to the conceptual engineering research program:

1. The explicatum is to be similar to the explicandum in such a way that, in most cases in which the explicandum has so far been used, the explicatum can be used; however, close similarity is not required and considerable differences are permitted. **(similarity)**
2. The characterization of the explicatum, that is, the rules of its use (for instance in the form of a definition), is to be given in an exact form, so as to introduce the explicatum into a well-connected system of scientific concepts. **(exactness)**
3. The explicatum is to be a fruitful concept, that is, useful for the formulation of many universal statements (empirical laws in the case of a nonlogical concept, logical theorems in the case of a logical concept). **(fruitfulness)**
4. The explicatum should be as simple as possible; this means as simple as the more important requirements (1), (2), and (3) permit. **(simplicity)**

Carnap (1962b, p. 7); cited by Cappelen, (2018, p. 11)

As for the first criterion, note that it does not follow from the fact that conceptual spaces are ultimately based on similarity judgments that the regions that, according to the space, represent concepts match concepts actually in use. Importantly, however, it is easy to check the descriptive adequacy of conceptual spaces empirically. After all, from such a space follow not only predictions about how people will judge the similarity of any pair of items representable in the space (in virtue of the fact that a conceptual space is *also* a similarity space); it also predicts whether people will *classify* any given item representable in the space as falling or not falling under a given concept in that space. For instance, given a conceptual color space (see, e.g., Jraissati and Douven 2018; Douven 2019), we can predict for *any* point in that space whether it will be classified as red, or blue, or green, and so on. While such predictions need not bear out, there is solid empirical support for the descriptive accuracy of most of the conceptual spaces known in the literature, meaning that, as a matter of fact, concepts as represented by those spaces do closely match concepts in actual use. That gives reason to believe that, by using conceptual spaces, we *are* satisfying Carnap’s similarity criterion for most concepts.

Likewise, we argue that concepts engineered through the CSF satisfy *exactness*. The procedure for constructing the spaces is explicit and uses well-known and well understood mathematical and statistical techniques, which have been shown to have wide scientific and practical applications. Furthermore, as Isaac (2021) points out, exactness can be understood in terms of three key aspects: consistency, precision, and unambiguity. Consistency corresponds to the absence of contradicting principles within the framework. In our analysis, the definition of concepts and their relations do not introduce any contradiction among concepts. By aligning as much as possible with participants’ categorizations, the construction of our conceptual space remains consistent with their perspectives while not conflicting with uncontroversial facts. Precision and unambiguity, on the other hand, are determined by descriptive accuracy and adequacy of the concepts. Through the application of the CSF, we guarantee precision and unambiguity by representing concepts in a multidimensional space that effectively captures the relevant features and relationships among them. Additionally, the use of clustering algorithms further ensures precision by organizing the data into distinct categories that align closely with participants’ judgments.

Furthermore, when it comes to the *fruitfulness* criterion the nature of the conceptual domain matters. For perceptual concepts, the fruitfulness of an analysis in terms of the CSF is determined either in terms of how it can help us understand the structure, properties, and sources of concepts related to color, sound, smell, and so on, or how it can help build better preforming artificially intelligent agents (see Gärdenfors, 2014 for discussion). For example, the CSF suggested a new, geometrical account of the vagueness of many perceptual concepts (Douven et al. 2013; Decock and Douven 2014; Douven and Decock 2017; Decock 2018), which in the meantime has received strong empirical backing both for color concepts and for shape concepts (see, e.g., Douven 2016, 2017, 2019; Douven et al., 2017). For social concepts, we suggest that an explication in terms of the CSF will be considered fruitful in case these representations can help us understand the structure, properties, and sources of concepts related to the social domain (in our case feminism), or if they give us some insight into where conflicts such as those described in the previous section are coming from. Whether the analysis to be offered further on will be fruitful in this regard is an empirical question, of course, which we will not try to answer in this paper.

The principles of cognitive economy that Douven and Gärdenfors (2020) propose could be argued to jointly imply that concepts are to be *simple*. Naturally, it only follows that concepts, as analyzed in the CSF, *are* simple if these principles are satisfied in actuality. But there is already substantial evidence that they are satisfied indeed (Regier et al. 2007, 2009; Jraissati and Douven 2017; Douven 2019; Zaslavsky et al. 2022).

All this builds on the assumption that it is possible to apply the CSF to social domains, something that, as discussed in the introduction, is considered far from obvious by researchers in cognitive science. Noteworthy applications of data reduction techniques in the social domain have been observed, however. For instance, multidimensional scaling (MDS) has been employed in studies on personality, such as Rosenberg and Jones (1972) and Jones and Rosenberg (1974). Eckes (1994) and Vonk and Ashmore (2003) used MDS and homogeneity analysis to create a geometrical representation of gendered subgroups. In a more recent study, Pattyn et al. (2013) employed a weighted Euclidean model to investigate the relevant dimensions of mental representation in the classification of pictures portraying people. Conversely, there is a long tradition in sociology that uses spatial representations to study social concepts. In developing our CSF study of feminism, we took important cues from the study of what sociologists call *social spaces*.

### Social spaces

Bourdieu (1979) explores the relationship between, on the one hand, the distribution of economic, social, and cultural assets and, on the other hand, patterns of practices and preferences in cultural, moral, and political matters. For this, he introduces a spatial construction of social positions—which he calls a *social space*—in which agents are represented as either dominant or subordinate. In this space, variables describe basic conditions of existence, such as economic capital (whether inherited or acquired) and cultural capital, including level of education, discipline, objectified cultural capital (books, records, paintings, etc.), and indices of social capital. The closer individuals are located to each other, the more similar they are with respect to the assets they have.

In his earliest work, Bourdieu is not explicit about how he constructs the social space. In later work, however, he notes that his spatial constructs are based on an aggregation of results from applications of a dimension-reduction technique very similar to MDS, viz., *Multiple Component Analysis* (Lebart et al. , 1984), to surveys carried out by the *Institut national de la statistique et des études économiques* (INSEE) from 1967 to 1972 as well as surveys from other researchers (see Bourdieu , 1989).

Bourdieu’s use of a social space has inspired much work in sociology, including Deauvieau et al. (2014), which in its turn inspired our study. Deauvieau et al. (2014) build on a branch of sociology that aims to define the relevant divisions for describing contemporary societies. Most often these debates are based on studies of objective differences between individuals or social groups in terms of living and working conditions as well as cultural and political practices. More rarely, subjective categorizations are taken as input. Deauvieau and colleagues’ study is of the latter kind. Specifically, these authors propose to investigate the nature of social cleavages on the basis of French people’s categorizations and to construct a French socio-professional space. To study French people’s representations of socio-professional divisions, they used an experimental paradigm designed to interrogate participants’ ordinary knowledge of such divisions. The paradigm derives from Boltanski and Thévenot’s (1983) work which presented a card game in which respondents are asked to comment on and rank cards representing individuals and their social characteristics according to their degree of similarity. Deauvieau et al. (2014) constructed thirty-three cards describing different “real” people randomly chosen from the *Fourth European Working Conditions Survey* (2005). Each of these cards represented a range of sociological information (as well as a fictional name) allowing participants to induce the person’s gender, age, and six socio-professional criteria. They asked 547 non-specialist French participants to arrange the cards into at most ten groups based on the social position of the individual described on the card. They then asked participants to name each of their groups and identify its most representative member. Based on these categorizations, Deauvieau et al. (2014) computed a similarity matrix, where pairs of cards were judged to be more or less similar based on how often participants put the two cards in the same group. Finally, they used multidimensional scaling to construct a similarity space.

In the next section, we will use Deauvieau et al. (2014)’s methodology to construct a social/conceptual space for feminism in France.

## Study

### Methods

#### Materials and procedure

While the materials in Deauvieau et al. (2014) consisted of anonymized individuals, in our study the materials consisted of the names of feminist authors and activists our participants could reasonably be expected to know. To generate the list of names, we used the CaFé corpus (see Section 2), from which we extracted all the occurrences of feminist figures cited by the interviewees, which amounted to 129 occurrences. We then selected all names of feminist figures that were cited more than once by more than one feminist activist. The idea was to obtain a list of names that are sufficiently well-known and salient to members of Parisian feminist communities so that they could be meaningfully categorized by our participants. This left us with 36 names, from which we removed figures that we hypothesized were frequently cited in the corpus because of its particularities. For example, since the corpus was originally constructed as part of a linguistics project, it contains many discussions about language and linguistic practices, so we suspected that linguists were over-represented in our names. This led to a final selection of 31 names of feminist figures which constituted our stimuli. For a list of all names, see the Supplementary Materials (though the names can also be gleaned from Table 4 or Figs. 3 and 5).

The study received approval from the ethics board of Paris Cité University (No 2022-38-bendifallah-burnett), and it was conducted online using the Qualtrics platform. The tasks were the same as in Deauviau et al. (2014)’s study. Specifically, participants were asked to sort as many of the 31 names that they recognized into at most nine groups. They could also make a tenth group for the figures that they did not know. Also as in Deauvieau et al. ’s study, they were asked to give a label to each of the groups and to identify the most representative member of each group. In the last part, we collected data about each participant (age, education, gender, sexuality, racial background) and their activism. We asked them to choose which feminist political identity term(s) they would primarily use to qualify their own feminism: “intersectional,” “materialist,” “queer,” “pro-sex,” “radical,” “decolonial” and/or “other(s).” If they chose “other(s),” they were free to write additional information.

#### Participants

Participants were recruited via a large French mailing list dedicated to researchers working in gender studies. Participants spent on average 35 minutes on the survey. They were compensated with a gift card worth € 15. A total of 81 participants completed the study. They identified mostly as white (68), under 35 (65), and highly educated (62 had a master’s degree or higher); they were almost all from France (75), with many from the Parisian region (29). Thus, the participants in our study formed a rather homogeneous social group. One area in which they varied concerns gender and sexuality. Most (56) participants identified as some feminine gender identity, but there was a significant number of participants whose gender did not respect the male/female binary (20), and a small number of participants with a masculine gender identity (5). The participants were almost equally divided between identifying as lesbians (26), heterosexual women (20), and bi- or pansexual people (24), with the remaining 11 participants having some other sexual identity, such as asexual or gay man.

### Results and discussion

Participants categorized the 31 feminist figures into at most nine groups. The constitutions of the groups depended first of all on how many of the names participants felt that they knew well enough to judge. Here, we found a fair amount of variation across our stimuli. For example, Simone de Beauvoir and Angela Davis were the only feminists who were known by all 81 participants, although Judith Butler, Olympe de Gouges, and Virginie Despentes were known by 80.^18^ This being said, all but four feminists (Gwenola Ricordeau, Joan Scott, Jules Falquet, and Michelle Perrot) were known by at least two thirds of the participants, and only Scott and Ricordeau were known by fewer than half of the participants (40 and 35, respectively). We therefore consider our technique of using the corpus to generate the stimuli as successful at providing a set of feminists who are relevant and salient to the community that we wanted to study.^19^

Participants varied with respect to how many categories they employed. While the design of our study allowed for nine categories, many participants used fewer. In fact, the average number of categories used in a single categorization schema was 7.5 (SD = 1.6). The fewest number of categories used was three (three participants), and 31 participants used the maximum number of nine. In addition to categorizations based on feminist political identity terms (*materialist*, *queer*, *intersectional*, *afrofeminist*, *decolonial*, *lesbian feminist*, *universalist*, etc.), participants made categorization schemas based on other kinds of considerations, as indicated by the category labels they used. These included profession (*politicians* vs. *journalists* vs. *academics*), temporality (*historical figures* vs. *contemporary figures*), political spectrum (*left wing* vs. *right wing*), and nationality (*French feminists* vs. *American feminists*). And, although there were some participants who made their groupings strictly according to feminist political identities—for example, participant 38 who proposed the categorization schema in Table 1—the majority proposed categorizations like participant 44, given in Table 2, which mix political identities with professions, temporality, platforms, and so on.

**Table 1:** Categorization of participant 38

Group	Feminists	Label
1	Badinter, Beauvoir, hooks	constructivist feminism
2	Butler, Wittig, Delphy	materialist feminism
3	Chollet, Schiappa, Hidalgo	liberal feminism
4	Woolf	white feminism
5	Coffin, Bourcier	queer feminism
6	Davis, Beyoncé	black feminism
7	Taubira, Gouges, Despentes, de Haas, Rich, yourself	intersectional feminism
8	other feminists	unknown

The size of the groups also varied, as Tables 1 and 2 show. Some groups are singletons and others contain many names. The feminist who was most often put in a class of her own is Beyoncé (25 participants), followed by *yourself*, where 12 participants created special singleton “me” categories (and three considered that their self was unknown).

**Table 2:** Categorization of participant 44

Group	Feminists	Label
1	Tuaillon, Beyoncé	feminists of the digital world
2	Badinter, Perrot, Gouges, Beauvoir	historical figures
3	Coffin, Rich, Despentes, yourself	radical lesbian feminists
4	Delphy, Dorlin	academic feminists
5	Schiappa, Fourest, de Haas, Hidalgo, Chollet,	
	Taubira	capitalist feminists
6	hooks, Davis, Vergès, Gay	decolonial feminists
7	Ricordeau	anti-carceral feminists
8	Butler, Preciado, Bourcier	queer feminists
9	other feminists	unknown

In addition to collecting self-identification data, we also asked participants to put themselves in a group. However, we did not incorporate this data in the construction of our similarity and conceptual spaces. Unlike the self-identification which focused on feminist political identities, participants’ self-categorization took into account various other criteria, including hobbies, temporality, profession, and topics of interest. For instance, participant 2 used a single category to categorize themselves as a “reader,” participant 7 classified themselves as a “contemporary intersectional,” participant 11 labeled themselves as an “academic feminist,” and participant 12 identified as a “thinker.”

#### Constructing a similarity space

As described in the previous section, the first step in constructing a conceptual space is to build a similarity space. Because the *yourself* item was unlike the others (being a kind of variable and often appearing in its own special “me” group), we discarded it from the analyses. Therefore, in what follows, we construct a similarity space in which we locate the 31 feminist names.

**Fig. 1:**
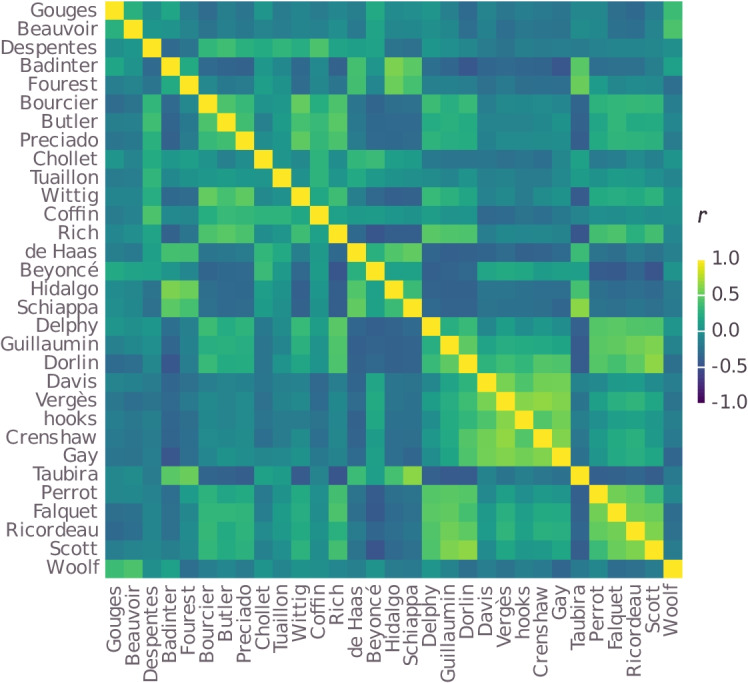
Plot of correlation coefficients among columns (or equivalently, rows) of Table 3

Following Deauvieau et al. (2014), we derive similarities from how often our participants group figures together in some group, which we can represent in a co-occurrence matrix, as shown in Table 3. As this table indicates, Olympe de Gouges and Simone de Beauvoir were grouped together by 53 participants; Gouges and Virginie Despentes were grouped together by 6 participants; and so on. Specifically, we start by calculating the correlation coefficients among the columns of this table (or equivalently, given that it is symmetric, among its rows). These are visually represented in Fig. 1. Next, we use the function $f(r) = \sqrt{2 - 2r}$, as for instance implemented by the cor2dist() function in the psych package (Revelle, 2023) for the statistical computing language R, to turn the correlation coefficients into distances.^20^

**Table 3:** Initial rows and columns of the co-occurrence matrix

	Gouges	Beauvoir	Despentes	Badinter	Fourest	Bourcier	Butler	$\ldots$
Gouges	0	53	6	18	3	1	2	$\ldots$
Beauvoir	53	0	6	23	2	2	6	$\ldots$
Despentes	6	6	0	3	3	14	17	$\ldots$
Badinter	18	23	3	0	37	3	2	$\ldots$
Fourest	3	2	3	37	0	9	2	$\ldots$
Bourcier	1	2	14	3	9	0	45	$\ldots$
Butler	2	6	17	2	2	45	0	$\ldots$
$\vdots$	$\vdots$	$\vdots$	$\vdots$	$\vdots$	$\vdots$	$\vdots$	$\vdots$	$\ddots$

Each entry indicates how many participants grouped the two corresponding feminists together

**Fig. 2:**
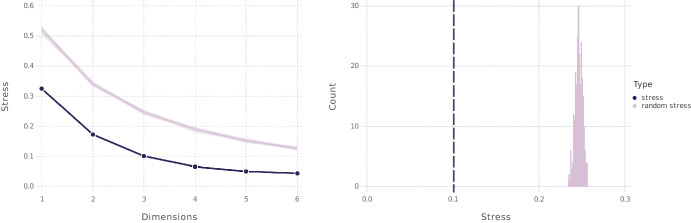
Left panel showing stress values of *n*-dimensional MDS models based on our data, for $n = 1,\ldots , 6$ (in blue), plotted together with stress values of 250 *n*-dimensional MDS models based on random similarity matrices (in purple); right panel showing stress values for the specific case of $n=3$

**Fig. 3:**
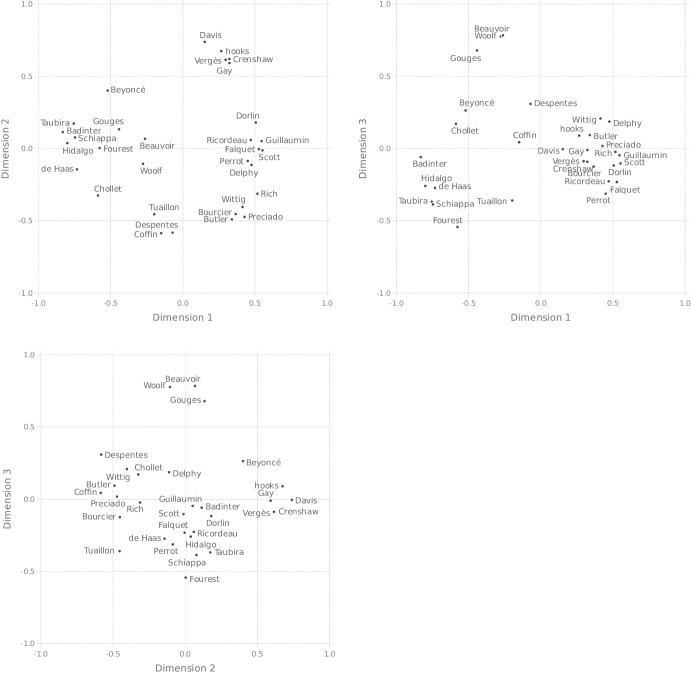
Pairs of dimensions of the three-dimensional similarity space based on the co-occurrence matrix partially shown in Table 3

To obtain the actual space, then, we apply an MDS procedure to these distances. More exactly, we used the SMACOF algorithm, implemented in the smacof package (Mair et al. , 2022) for R, to construct spaces with numbers of dimensions ranging from 1 to 6. As already briefly mentioned in Section 3.1, goodness of fit for MDS output is commonly measured in terms of stress, where the stress value indicates how closely the distances between objects in the configuration resulting from the MDS procedure match the similarities between the items underlying the distance matrix. The left panel of Fig. 2 shows the stress values for the results of the various MDS solutions we obtained. The figure also allows us to compare these values, for each relevant number of dimensions, with the stress values associated with 250 other MDS solutions with the same number of dimensions, where these other solutions were based on random similarity matrices. According to some authors (e.g., Dexter et al. 2018), this gives a better indication of how good an MDS solution is than its associated stress value considered in isolation. In the figure, it is seen that stress diminishes sharply (and thus goodness of fit increases sharply) when we move from one to two dimensions, and then substantially from two to three dimensions, but less so when we move to higher dimensions. Also, we see that the three-dimensional model has a stress value of .1, which is considered good. The right panel of Fig. 2 shows specifically for this model that its fit is significantly better than what could be expected by chance. We thus decided to go with the three-dimensional model, which is visualized in Fig. 3.^21^

As a quick sanity check, one can observe that, for instance, Olympe de Gouges, the 18th century feminist author and revolutionary, is much closer to Simone de Beauvoir, whose foundational work laid the groundwork for second wave feminism, than to Sam Bourcier, the contemporary queer scholar and transfeminist activist. Likewise, Bourcier is much closer to Judith Butler, with whom he shares a political orientation, than Butler, American queer academic, is to Elisabeth Badinter, one of the main figures of French universalism. This is all exactly as should be.

More importantly, however, we explained in Section 3.1 that, in an MDS analysis, one not only aims at a low-dimensional and well-fitting representation of the input data, the dimensions of the space in which the data are represented should also be interpretable. For the space that came out as best of the MDS procedure, the interpretation of the dimensions seems rather straightforward. The first dimension has the politicians Anne Hidalgo, Marlène Schiappa, and Christiane Taubira, along with the politically active writers Elisabeth Badinter and the activist Caroline de Haas, on one end, and the academics, such as bell hooks, Kimberlé Crenshaw, Jules Falquet, Judith Butler, Sam Bourcier, and Paul Preciado, on the other end. In the middle, we find authors, journalists, and artists, such as Beyoncé, Victoire Tuaillon, and Virginie Despentes. Thus, a plausible interpretation of the first dimension is that it is “profession” related, plausibly indicating degree of (explicit) political engagement, with the two extremes being “politician” and “academic.” Another plausible interpretation concerns radicality ranging from institutional feminism to a more revisionary kind of feminism. Institutional feminism focuses on creating change within existing institutions and systems. Politicians would be good representatives of this kind of feminism but this interpretation would also allow Beyoncé and Chollet, who are not politicians but are closer to this group, to be interpreted on this continuum. On the other hand, revisionary feminism seeks to challenge power dynamics by incorporating intersectionality and a more nuanced understanding of gender identity. This includes intersectional, materialist, and queer figures that can be found on the other end of the dimension.

The second dimension depicts scholars who work on sexuality (and gender), particularly in queer studies (Butler, Preciado, Bourcier, Wittig) on one end, and scholars whose work investigates questions related to race and gender (hooks, Davis, Crenshaw, Vergès) on the other. The politicians, artists and journalists are in the middle, as are the lesser known academics (Ricordeau, Scott, Perrot, Falquet) and major figures of French materialist feminism such as Christine Delphy and Colette Guillaumin. Note however, that Dorlin, Guillaumin and Ricordeay are closer to Vergès, Crenshaw, Davis, and hooks, than Delphy is, possibly reflecting their focus on race. Thus, we consider that dimension 2 can be described as representing a *focus on sexuality–focus on race* continuum.

The third dimension is a bit harder to interpret. It is characterized by a large separation between Olympe de Gouges, Simone de Beauvoir and the early 20th century writer Virginia Woolf on one end, and all the other feminists on the other. Given that Gouges, Beauvoir, and Woolf are the only figures in our stimuli whose most famous activities took place before the 1960s, we suggest that dimension 3 corresponds to some kind of “temporality” scale. Interestingly, there do not appear to be clear temporal distinctions within the large cluster of contemporary feminists. Within this large group, the feminists do not appear to be ordered by age: Joan Scott and Michelle Perrot are from an older generation than Beyoncé and Mona Chollet, yet they appear further away from Beauvoir, Gouges, and Woolf on the third dimension. More generally, the participants in our study do not seem to be distinguishing between different “waves” of feminism: Judith Butler, Monique Wittig, and Virginie Despentes are all practically at the same point on dimension 3, yet Wittig is generally considered to be a “second wave” feminist, while Butler and Despentes are important figures of the “third wave” in France and abroad (Schaal , 2017). Thus, the relevant temporal distinction to our participants appears to be something like *historical figures* (mid 20th century or before) versus *contemporary figure* (late 20th/early 21st century).

In short, the above considerations suggest that our best space has a politicians–academics dimension, a sexuality–race dimension, and a historical–contemporary dimension.

#### Partitioning the space

Recall that a conceptual space consists of both a similarity space and a partition of that space into cells, which are supposed to represent a family of concepts. As previously explained, the most common way to partition a space is through first identifying a relevant set of prototypes and then letting those generate a Voronoi tessellation. In our study, as a way of trying to obtain prototypes, we asked participants to indicate which feminist they considered to be the most representative of the group, for each group that they constructed. But this procedure failed to yield useful results, possibly because participants used different categorization strategies but possibly also because we are dealing with a conceptual domain for which the notion of prototype is unhelpful (see note 14).^22^

Thus, instead of using prototypes to define the concepts in the similarity space (i.e., going “top down”), we decided to go “bottom up” and use the clustering approach also mentioned in Section 3.1 to identify which feminists are to fall under the same concept. In particular, we used the PAM clustering algorithm, implemented in R in the cluster package (Maechler et al. , 2022), which, as previously said, is a more robust version of the older *k*-means clustering and, like it, aims to partition a number of items into *k* clusters in such a way that, broadly speaking, within-cluster similarity and across-cluster dissimilarity are jointly maximized.

**Fig. 4:**
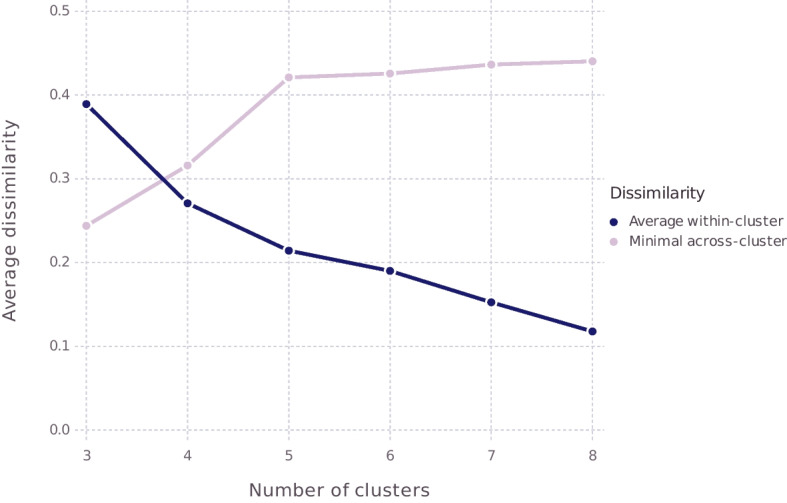
Average dissimilarity within a cluster (blue) and minimal dissimilarity between clusters (purple) for PAM solutions, with number of clusters going from 3 to 8

**Fig. 5:**
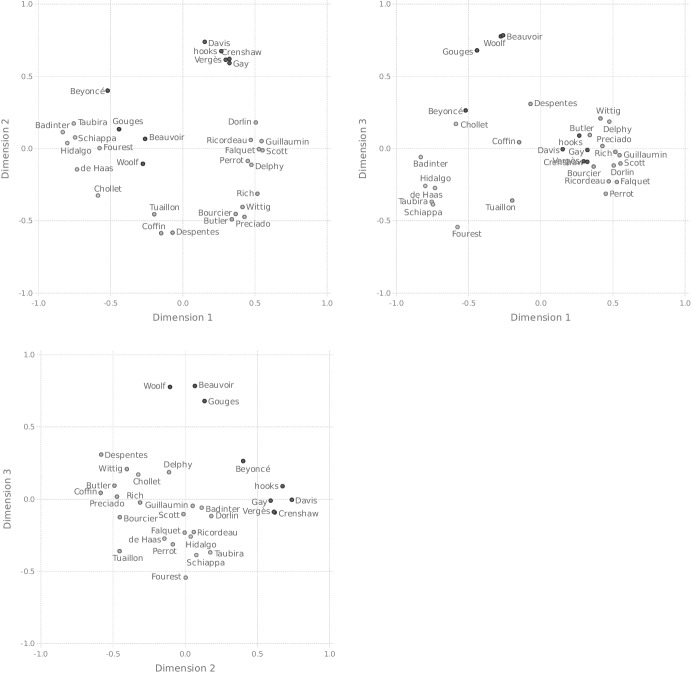
Pairs of dimensions of the conceptual space of French feminism

It was also noted that the number of clusters needs to be pre-specified, which is to say that the algorithm will not pick that number for us. The standard way to determine the optimal number of clusters is to run the algorithm for a range of numbers of clusters and then compare the goodness of fit of the various resulting solutions, which in the case of the present algorithm can be done by considering how similar the items in each of the clusters are to the medoid of that cluster, which measures the within-cluster similarity (as explained in Section 3) as well as by considering the minimal dissimilarity between items in different cluster, which measures the across-cluster dissimilarity. Figure 4 plots both the within-cluster similarity and across-cluster dissimilarity for PAM applied to the coordinates of the feminist figures in our best similarity space, fitted on the basis of the responses from our participants, for number of clusters going from 3 to 8. In general, increasing the number of clusters yields better fit, which could suggest putting each item in its own cluster. However, that would yield a completely uninformative clustering. Ideally, then, what one finds in the kind of plot shown in Fig. 4—a so-called scree plot—is a discernible “elbow,” indicating that, for some given *n*, by going from *n* clusters to $n+1$ clusters we obtain a notable improvement in fit but then by going from $n+1$ clusters to $n+2$ clusters, much less improvement is achieved, in which case we would go with $n+1$ clusters. We do find this in Fig. 4, at least for across-cluster dissimilarity, where we get a big improvement by going from 3 to 4 cluster and then again by going from 4 to 5 cluster, but then going to 6 or more clusters hardly improves the across-cluster dissimilarity. We thus decided to to go with a partition into five clusters. The result is shown in Table 4, along with the list of the labels that were most often used to the members of these groups. Figure 5 depicts the conceptual space this clustering gives rise to.^23^

We see that the first cluster consists of Sam Bourcier, Judith Butler, Alice Coffin, Virginie Despentes, Paul Preciado, Victoire Tuaillon, and Monique Wittig. As mentioned above, Butler, Boucier, and Preciado are the main representatives of queer feminism in France, and so, unsurprisingly, the most commonly used label for the members of this group was *queer* (156 times). *Queer* was actually the clear winner for this category, with the next most frequent label being *lesbian* with only 68 occurrences for the members of this cluster.

The second cluster consists of the politicians Elisabeth Badinter, Caroline Fourest, Anne Hidalgo, Marlène Schiappa, Christiane Taubira, the activist and consultant Caroline de Haas and the journalist Mona Chollet who is primarly known for her books exploring feminist perspectives. Interestingly, the most common label applied to members of this group is *not (really) feminist* (101 times), but this is closely followed by the profession *politician* (91 times). The third most common label is *racist*, but this is less common (only 56 occurrences).

**Table 4:** Clusters of feminist figures resulting from applying PAM, with five clusters, to the distances derived from the co-occurrence matrix shown in Table 3

Cluster	Members	Most frequent label(s)
1	Bourcier, Butler, Coffin, Despentes,	queer (156)
	Preciado, Rich, Tuaillon, Wittig	
2	Badinter, Chollet, Fourest, de Haas,	not really feminist (101)
	Hidalgo, Schiappa, Taubira	politicians (91)
3	Beyoncé, Beauvoir, Gouges,	historical figures (125),
	Woolf	mainstream feminists (35), authors (26)
4	Crenshaw, Davis, Gay, hooks,	intersectional (153),
	Vergès	afrofeminist (79), decolonial (66)
5	Delphy, Dorlin, Falquet, Guillaumin,	academics (90), materialists (74)
	Perrot, Ricordeau, Scott	

The third cluster consists of the three figures who were distinguished by the third dimension of our space, Simone de Beauvoir, Olympe de Gouges, Virginia Woolf, along with Beyoncé. By far, the most common label for this group is *historical figures* (125 occurrences), followed by *mainstream feminists* (35) and then *authors* (26). At first, it looks like we have the same situation as with Group 2: the same group of feminists is labelled differently by different participants. However, the situation with Group 3 is actually quite different: all the occurrences of the label *historical figures* are applied to Beauvoir, Gouges, or Woolf, and almost all the occurrences of the label *mainstream feminists* are applied to Beyoncé (26/35). As just explained, this is due to the “granularity” of our clustering: in the clustering solution for $k=6$, we find that Beauvoir, Gouges, and Woolf form a separate cluster, with Beyoncé and Chollet ending up in a cluster with Coffin, Despentes, and Tuaillon.

Group 4 consists of five feminists whose work often focuses on gender and racial issues: Kimberlé Crenshaw, Angela Davis, Amandine Gay, bell hooks, and Françoise Vergès. By far, the most common label for the members of this group is *intersectional* (153 occurrences). This being said, this group is also frequently described as *afrofeminist* (79 times) and *decolonial* (66 times). Again we see a multitude of terms applied to the same group of people (like Group 2), and this result provides insight into the source of the conflict described in (8). Recall that, in that passage, two feminists disagreed whether the label *intersectional* is synonymous with *afrofeminist*. Our results show that for many people, it is not, but for some, it is.

Finally, Group 5 consists of the feminist academics Christine Delphy, Elsa Dorlin, Jules Falquet, Colette Guillaumin, Michelle Perrot, Gwenola Ricordeau, and Joan Scott. Unsurprisingly, the most common label is *academics* (90 times), but this is followed closely by *materialists* (74 times). Once more we see participants using multiple labels applying to the same figures. In this case, it is a more similar situation to Group 2, where participants varied between a “profession” based categorization and a “political identity” based categorization of the same figures, rather than to Group 3 where the two labels applied principally to different subgroups.

Note that studying the labels given to the categories by our participants suggests somewhat alternative interpretations of the first two dimensions of our similarity space. Specifically, based on the labels, it would also be reasonable to interpret the politicians–academics dimension as a *non-feminists versus feminists* dimension and to interpret the sexuality–race dimension as a *queer versus intersectional* dimension, with the materialist feminists forming a sort of midpoint on that dimension.

#### Discussion

The results of our conceptual spaces analysis give us a new understanding of how feminism in France is structured, at least in the minds of our participants. Using the CSF, we have discovered that, in the minds of feminists on an academic mailing list, there is a strong relationship between being an academic and being considered a (real) feminist. We also discovered that, within the feminist academic space, the salient oppositions are *intersectional/focusing on race* versus *queer/focusing on sexuality*. We observe that this opposition does not correspond to ones that are generally identified in the feminist domain (including by speakers in the CaFé corpus): *intersectional* versus *universalist*; *anti-racist* versus *racist*; *queer* versus *materialist*; and so on. This is an interesting finding, because these conflicts do appear in the language found in the group labels: members of the *politicians/not feminists* group are sometimes referred to as *racists* and *universalists* by participants. But, when the general patterns of categorization are taken into account, the polar opposite of bell hooks turns out not to be Marlène Schiappa, but Judith Butler. Likewise, the polar opposite of Butler is hooks, not the materialist Christine Delphy (who occupies a “neutral” middle position on the relevant dimension). Finally, we also discovered that, at least with this particular set of stimuli, the pertinent temporal distinction is between feminists who were active before the 1960s and those who made their most notable contributions afterwards. The third dimension thus defines an age of “contemporary feminism” for our participants, most of whom are under 35. Because of these new insights, we argue that our application of the CSF to this complex social domain has been enlightening, and we believe that this opens the door to more investigations of social concepts using this methodology in the future.

With respect to conceptual engineering: we saw that, using PAM clustering to partition the three-dimensional Euclidean similarity space into five categories leads to a plausible explication of the meanings of feminist political identity terms. In this approach, using the term *queer* as an identity term—as in *Elle est queer* (“She is a queer feminist”)—involves locating a person in the region of our conceptual space where Judith Butler, Sam Bourcier, Monique Wittig, and Paul Preciado are also located, or, alternatively, communicating that they have a high value on the *feminism* dimension, and a low value on the *queer–intersectional* dimension. Saying that someone is a *matérialiste* communicates that they are located in the same region of conceptual space as Christine Delphy, Colette Guillaumin, Adrienne Rich, Jules Falquet, among other people: they have a high value on the *feminism* dimension, and no extreme value on the *queer–intersectional* dimension. Identifying someone as *intersectionnelle* locates them in the same part of the space as Kimberlé Crenshaw, Angela Davis, bell hooks, Amandine Gay, and Françoise Vergès, specifically in an area with a high value on both the *feminism* and *queer–intersectional* dimensions. Finally, identifying someone as a *féministe* (tout court) involves placing them in a space that has a high value on the *feminism* dimension, a space that is devoid of politicians.

## General discussion

In this paper, we addressed the question of whether the conceptual spaces framework could be useful to the project of conceptual engineering. We identified feminist political identity terms, and feminism in France more generally, as a conceptual domain that might benefit from engineering, particularly Carnapian explication. In our quest for explication through conceptual spaces, we also addressed the question of whether the CSF could meaningfully be applied to social domains. We believe that the results reported in this paper constitute an affirmative answer to both of these questions. The similarity space that we constructed using the CSF methodology has interpretable dimensions, and the clustering algorithm yields categories to which labels taken from participants’ answers can be plausibly assigned (“plausibly” in light of our knowledge of feminism in France). We argued that these categories can constitute explicated versions of political identity terms such as *intersectional*, *queer*, *materialist*, and even *(not) feminist*. Even if these explicated concepts are not widely adopted (at least not yet), our results should be of interest for French feminist scholars and activists, since they provide valuable new information about how the feminist scene is structured in the mind of (a sample of) those who participate in it. Because of this, we consider that our work has been fruitful (in the Carnapian sense) and therefore that the CSF can indeed be a useful tool for conceptual engineering. Our conclusions are in line with those of De Benedetto (2020), who argues that conceptual spaces can be useful for the engineering of scientific concepts. We have shown that they apply equally to at least some social domains.

Our paper was strongly motivated by the observation that, insofar as participants to debates about feminist issues in France were clear about terms that are seemingly central to these debates, the concepts they associate with these terms appear to be poorly aligned. We proposed a specific way of constructing conceptual spaces as a method for generating a single point of alignment which possesses various attractive features. For instance, it was mentioned that PAM clustering is guaranteed to create a Voronoi tessellation, and the cells of Voronoi tessellations are provably convex (Okabe et al., 2000, p. 58), where convexity—it was seen—can be conceived as a requirement of cognitive economy. Moreover, as Douven and Gärdenfors (2020) note, the same clustering method yields results that are well-formed in the sense of Regier et al. (2007), which is another principle of cognitive economy. (Well-formedness, in this sense, also amounts to a joint maximization of within-cluster similarity and across-cluster dissimilarity, as defined in Section 3.1.) Most importantly perhaps, all of this is achieved while preserving the original concepts as much as possible.

Naturally, one could take a more revisionary stance and then find the concepts as represented in Fig. 5 to be still suboptimal. For example, one of the most interesting results of our study is that members of the feminist community that we studied perceive an opposition between focusing on sexuality versus focusing on race. This could be reason for thinking that the family of concepts in Fig. 5 is not entirely satisfactory. After all, it is factually incorrect to think that bell hooks or Angela Davis never consider questions of sexuality or that Judith Butler never considers questions related to race. This opposition arises in the conceptual space because these scholars’ works on race and sexuality respectively have been less influential, and this is reflected in our participants’ judgments. For this reason, it is possible that the CSF is relevant for only a kind of conceptual engineering that aims to remain as close as possible to the original concepts. More extreme engineering may have to use other methods.

It would in fact be unreasonable to expect that the CSF will give results that are satisfactory in every possible respect, at least when applied to social domains, given that human beings, and our representations of them, are complex in ways that color chips, sounds, or simple shapes are not. For example, someone like Monique Wittig is many things: an academic, an author, a French citizen, a lesbian, deceased, someone who analyzes gender as a social class, someone whose work focuses on sexuality, and so on. And participants vary with respect to how much they know about her life and which aspects of her work or life are most important to them. We suggest that the complexity of how we conceptualize people, which is at the heart of the social world, provides a serious challenge to a simple application of the CSF and at least needs to be appreciated by researchers aiming to further the scope of the CSF to domains beyond the perceptual.

Another potential challenge for using the CSF to explicate political identity terms comes from variation in how participants apply these terms to themselves versus to others. Recall that, in the CSF, the basic concepts partition the similarity space. As shown in Table 4, all figures appear in exactly one cluster. As a consequence, in our explicated concepts, identifying as (for example) *queer* implies not identifying as *materialist* or *intersectional*, since the regions picked out by each political identity term are disjoint. This structure corresponds well to our data where participants are asked to categorize other people: as Table 4 shows, each political identity term is very strongly associated with exactly one group. However, our participants appear to use these terms differently when they describe their own identities. When we asked participants to choose which terms they identified as, 24 responded that they identified as **both** queer and materialist. Far from being unusual, self-identification with multiple terms was actually the norm in our study: only 11 out of 81 participants selected a single term, and the average number of terms selected was three. *Intersectional* was selected by all but 10 of our participants, making it a kind of “default” political identity term. It is possible that the result that participants are much more lenient with their self-identification than they are with how they identify others is a product of our task: we allowed participants to select multiple labels for themselves but required that they name each group with a single label. We think there is something to this idea, since when we asked participants to place themselves in a group, only 10 of them put themselves in a group with *intersectional* in its name. This is comparable to *lesbian feminists* (13 participants) and *queer feminists* (10 participants). However, multiple identification cannot be uniquely due to the nature of our task because participants in the corpus interviews also spontaneously identify with multiple terms, as shown by the quotation from Speaker 32 in (6). The idea that these feminist political identity terms are applied more strictly to others than to the self could also be at the source of the conflict surrounding how strongly the meaning of *intersectional* is associated with afro-descendants. This includes the differing perspectives of afrofeminist activists and those of other heritage on how the term should be applied and understood. Thus, we conclude that applications of the CSF to the social domain and its incorporation into the conceptual engineering research program have a lot of potential, but there are still some outstanding methodological and theoretical challenges.

Finally, we would like to point out a possible benefit of the method of conceptual engineering that so far has not been fully appreciated in the literature, to wit, what we might broadly indicate as *helping to foster a conceptual consensus*. To explain what we mean by this, first note that, for a language to be a successful tool of communication, its users must mean, more or less, the same thing when they use a word. That is to say that the users’ concepts must be aligned with each other, at least broadly. Gärdenfors (2014) shows how such alignment can be the outcome of a coordination process in which different users of a language keep adjusting their concepts until they achieve a consensus, or at least approximately so.

The data from the CaFé corpus suggests that there is little agreement among participants to debates about feminism on the meanings of what would appear to be terms central to those debates. What is more, some participants may have unclear understandings of at least some of those terms. Naturally, given enough time, an agreement could emerge as a result of the aforementioned kind of coordination process. This consensus may help clarify the relevant concepts to all involved in feminist debates.

We hope that this paper has shown how conceptual engineering may be able to help here. One way to conceive of our analysis is as a method for identifying, clarifying, and regimenting the use of, a set of concepts at the core of today’s debates about feminism, where the method tries to be as conservative as possible, aiming to be in line with current usage of these terms as much as possible, by finding some sort of common denominator in how different participants to the said debates use these terms already.

## Footnotes

1 The question of the relevance of conceptual spaces for conceptual engineering has been discussed with respect to color concepts by Decock (2021). As discussed below, there are reasons to believe that perceptual domains, like the color domain, and socio-political domains, like feminism, are very different. Thus, even though Decock’s conclusions were somewhat negative, it is not clear that they transfer outside perceptual domains.

2 We are emphasizing “strongly” here because, as an anonymous referee noted, there is still some reliance on pre-theoretical judgments in the methodology we are proposing as well, most notably perhaps when it comes to interpreting the dimensions of the metric spaces that are central to our methodology (for details, see below).

3 For a notable exception, see Verheyen and Peterson (2021), which applies the CSF to the moral domain.

4 It is widely accepted that intersectionality has been coined by Crenshaw (1989). It is worth noting that some discourses referring to this methodology preceded Crenshaw’s publication. See, for instance, Combahee River Collective (1983), hooks (1984), King (1988), Spelman (1988).

5 Note that *queer* in France more often refers to a political orientation within feminism, rather than a sexual orientation, as it tends to do in North America (see Abbou and Burnett, 2022b).

6 So me I feel queer, I put myself with the queer feminists. I use the term which is a kind of activism and which for me actually covers all the realities, and which is often viewed by the mainstream as glitter, I think.

7 At university where most people were materialists, I learned that a bit and it interests me more and more, and a lot more now, so I don’t know if I would call myself a “materialist feminist,” or I wouldn’t call myself “queer” in any case. That’s for sure.

8 Inspired by Queer theory but the currents that try to draw a link between queer feminism and materialism. It would be a bit in this and intersectional, well, trying to be intersectional, because it’s easy to say “I’m intersectional,” but my feminism aims to be queer and intersectional.

9 When I met in Lyon people who were interested in the relationship between materialism and queer feminism, and so we often had debates $\ldots$ because I was part of a community that defined itself as “queer,” people who more and more defined themselves as queer. It’s hard for me to see what exactly that term covers.

10 For a long time I defined myself as a “materialist feminist” without knowing very well what was behind this term besides what we were saying between friends.

11 I think that I put issues of class and race at the heart of my feminism, but I have the impression that everyone says this but no one puts the same think behind it $\ldots$ a couple years ago, I would have given all the terms, actually, I would have said “my feminism is materialist, intersectional, decolonial,” etcetera and actually I realize that, when one uses those terms, it’s also a way of masking what one thinks. **Interviewer:** to maintain the vagueness.

12 My impression is that it’s a term that is claimed because it is obvious that feminism should be intersectional for feminists, but in fact it is instrumentalized to avoid thinking about relations of domination that are not necesarily linked to gender. In its use in that way, it annoys me a bit. It’s a bit like saying “well me I’m feminist so I don’t need to think about relations that I have with –” you see $\ldots$ of saying “well actually we’re intersectional so it’s not a problem if you’re racialized,” and without really reflecting on the relations of domination that exist. So yeah it’s a term that is a bit hollow in its use, but for sure for me it’s something that is absolutely essential.

13 We said that we were an intersectional association and actually the afrofeminists, there was one activist chick who wrote to us to say “yeah, you can’t say that it’s intersectional because that’s a word that’s reserved for afro-descendents” but OK apparently if you use it as an adjective actually that implies that you’re actually an afrofeminist association.

14 Clustering would seem the right approach to building conceptual spaces from the perspective of exemplar theory, according to which people categorize items by comparing them with previously encountered ones rather than with prototypes; (see, for instance, Nosofsky 2011). Whereas it is common in the literature to present prototype theory and exemplar theory as rival approaches to representation, we believe this to be a mistake. It makes perfect sense to hold that some concepts have prototypes while others do not. For instance, people tend to have no difficulty identifying prototypes of basic color concepts (see, e.g., Douven, 2019). By contrast, one would seem hard pressed to identify the prototypical Renaissance painting or the prototypical classical piano sonata. (See, e.g., Douven, 1999) on how identifying a work of art as belonging to a certain school or era is more plausibly thought to proceed by comparing its features to those of works of art one is already familiar with.)

15 Or, in the case of multi-domain concepts (like the concept of apple, for instance, which requires color space, taste space, a shape space, and more, for its representation), as *sets* of such regions; for details, see Gärdenfors (2000, ch. 4).

16 Or sets of convex regions, in the case of multi-domain concepts; see note 15.

17 Not all proponents of the CSF are convinced that convexity is even necessary for naturalness (e.g., Strössner, 2022). It is to be noted, however, that the concerns that have been raised about the convexity requirement are based on spaces that have been drawn for illustrative purposes only and lack proper empirical support.

18 See the Supplementary Materials for how many times each feminist was categorized in some group other than “unknown.”

19 At the same time, we recognize that the still relatively great number of “unknowns” in our response requires some special attention, as they might skew the results. For this reason, we reran the analysis to be reported in the following on a reduced dataset, leaving out the upper quartile of “least known” feminists. Because qualitatively the re-analysis led to nearly identical outcomes, it is reported only in the Supplementary Materials.

20 Ideally, we would have measured similarities directly, by eliciting similarity judgments for each pair of names in our materials. However, this would have required participants to make $$\left( {\begin{array}{c}31\\ 2\end{array}}\right) =465$$ judgments (supposing order of appearance of the names to make no difference; otherwise, the participants would have had to make 930 judgments). That would have made the survey excessively long (as well as excessively expensive). Hence, we opted for Deauvieau and colleagues’ design, as providing a quick and inexpensive alternative. However, the data thus obtained can be used as a basis for an MDS procedure in more than one way. Instead of using correlations between co-occurrence profiles, we could have used co-occurrence frequencies directly as proxies for similarities. As far as we are able to see, it is not a priori that one option is to be preferred over the other. Therefore, we have conducted an analysis on the basis of the second option as well, which is presented in a separate Jupyter notebook contained in the Supplementary Materials. This will allow interested readers to verify that, at least in the present case, the two options give virtually the same results. (Thanks to Steven Verheyen for suggesting the alternative analysis to us.)

21 Figure 3 presents two-dimensional plots of each pair of dimensions. Readers are encouraged to download the Jupyter notebook that is part of the Supplementary Materials. This contains a three-dimensional plot of the similarity space that can be rotated, which allows one to get a much clearer impression of the location of the items in the space.

22 In this connection, it is also worth mentioning that although, as previously said, Deauvieau et al. (2014) collected data on typicality, these data played no role in their analysis and in fact are not even reported in the paper.

23 Again, there is a three-dimensional plot of the conceptual space in the Supplementary Materials. Rotating it in the notebook will give a better understanding of the structure of the space.
